# Benzene-1,3,5-triyl tris­(2,2-dimethyl­propanoate)

**DOI:** 10.1107/S1600536809050028

**Published:** 2009-11-28

**Authors:** Alan H. Haines, David L. Hughes

**Affiliations:** a School of Chemistry, University of East Anglia, Norwich NR4 7TJ, England

## Abstract

In the title compound, C_21_H_30_O_6_, the three acet­oxy groups are essentially planar with their normals rotated by −57.75 (4), −62.36 (4) and 63.36 (4)° from the normal to the mean plane of the C_6_ ring. The arrangement of carbonyl groups around the ring is of two groups ‘up’ and one ‘down’, and the propeller-style arrangement of substituent groups is spoiled with the plane of the ‘down’ group twisted in the opposite direction; all the C_ring_—O—C—CMe_3 _conformations are *trans*. In the crystal, aromatic π–π stacking occurs [centroid–centroid separation = 3.320 (1) Å]; the other main inter­molecular inter­action is a C—H⋯π-ring contact on the opposing side from the overlapped ring pairing; there are no short C—H⋯O contacts.

## Related literature

For our previous studies in this area, see: Haines & Hughes (2007 ▶); Haines *et al.* (2008 ▶, 2009 ▶). For a related structure, see: Haines & Hughes (2009 ▶). For furhter synthetic details, see: Hegetschweiler *et al.* (1990 ▶).
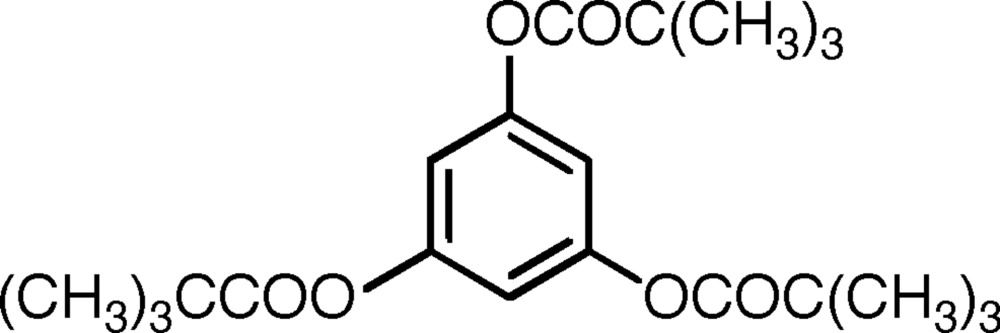


## Experimental

### 

#### Crystal data

C_21_H_30_O_6_
*M* *_r_* = 378.45
Triclinic, 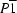

*a* = 9.4812 (3) Å
*b* = 10.6885 (3) Å
*c* = 11.4799 (3) Å
α = 91.138 (2)°
β = 97.649 (3)°
γ = 111.624 (3)°
*V* = 1068.87 (5) Å^3^
*Z* = 2
Mo- *K*α radiation
μ = 0.09 mm^−1^
*T* = 140 K
0.65 × 0.32 × 0.15 mm

#### Data collection

Oxford Diffraction Xcalibur 3/CCD diffractometer
Absorption correction: multi-scan (*CrysAlisPro RED*; Oxford Diffraction, 2008 ▶) *T*_min_ = 0.920, *T*_max_ = 1.059
26503 measured reflections
6205 independent reflections
4605 reflections with *I* > 2σ(*I*)
*R*_int_ = 0.038

#### Refinement

*R*[*F* ^2^ > 2σ(*F* ^2^)] = 0.047
*wR*(*F* ^2^) = 0.132
*S* = 1.08
6205 reflections
244 parameters
H-atom parameters constrained
Δρ_max_ = 0.65 e Å^−3^
Δρ_min_ = −0.34 e Å^−3^

### 

Data collection: *CrysAlisPro CCD* (Oxford Diffraction, 2008 ▶); cell refinement: *CrysAlisPro RED* (Oxford Diffraction, 2008 ▶); data reduction: *CrysAlisPro RED*; program(s) used to solve structure: *SHELXS97* (Sheldrick, 2008 ▶); program(s) used to refine structure: *SHELXL97* (Sheldrick, 2008 ▶); molecular graphics: *ORTEP-3* (Farrugia, 1997 ▶); software used to prepare material for publication: *SHELXL97*.

## Supplementary Material

Crystal structure: contains datablocks I, global. DOI: 10.1107/S1600536809050028/hb5166sup1.cif

Structure factors: contains datablocks I. DOI: 10.1107/S1600536809050028/hb5166Isup2.hkl

Additional supplementary materials:  crystallographic information; 3D view; checkCIF report

## Figures and Tables

**Table 1:** Hydrogen-bond geometry (Å, °)

*D*—H⋯*A*	*D*—H	H⋯*A*	*D*⋯*A*	*D*—H⋯*A*
C13—H13*B*⋯*Cg*1^i^	0.96	2.82	3.7608 (16)	168

Symmetry code: (i) 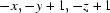. *Cg*1 is the centroid of the C1–C6 ring.

**Fig. 1.**
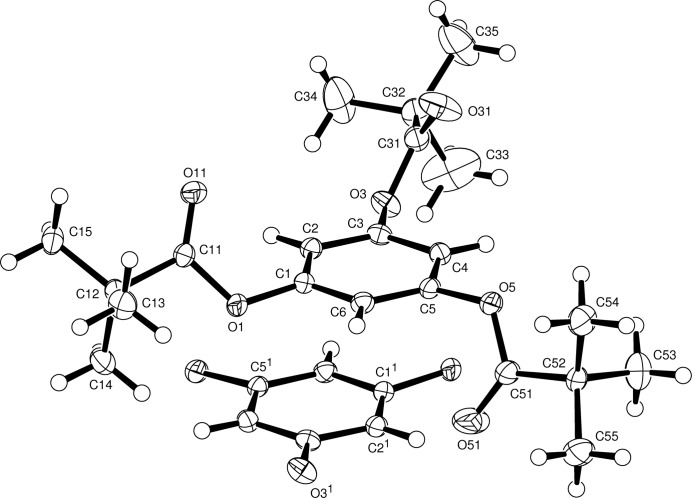
The molecular structure of (I). Part of a related, neighbouring molecule is also shown; C(21) is directly under the ring C(1–6). Displacement ellipsoids are drawn at the 50% probability level.

## supplementary crystallographic information

### Comment

Structural factors which enhance the solubility of organic compounds in liquid carbon dioxide are difficult to identify, but a knowledge of these is important in view of the possibility of using liquid carbon dioxide as an environmentally acceptable, cheap, safe and readily available alternative to replace organic-based solvents in the development of so-called "green chemistry". Previous studies (Haines *et al.*, 2008) have shown that certain types of acyl group promote the solubilities of per-acylated *D*-glucopyranose derivatives in liquid carbon dioxide; in particular trimethylacetyl groups promoted solubility, their effect being comparable to acetyl groups and superior to dimethylacetyl groups. In searching for an explanation for solubility differences in this series based on differing intermolecular forces in the solid state, we conducted crystal structure studies on the compounds (Haines & Hughes, 2007), but the results indicated no substantial difference in such intermolecular forces.

Measurement of solubilities in liquid carbon dioxide of 1,3,5-triacetoxybenzene (2) and substituted derivatives, *viz* 1,3,5-tris-(dimethylacetoxy)benzene (3) and 1,3,5-tris-(trimethylacetoxy)benzene (1), chosen in an attempt to separate the effects on solubility of the number and structure of peripheral substituents in compounds of similar overall molecular dimensions to the carbohydrate derivatives, showed no major differences (Haines, *et al.*, 2009, unpublished results) and prompted an investigation of their crystal structures in order to compare intermolecular interactions in these compounds.

The stucture of the title compound, (1), is shown in Figure 1; compound 2 is described in the preceding paper (Haines and Hughes, 2009). Unfortunately, of the crystals of 3 grown under a variety of conditions, none gave acceptable diffraction patterns, all showing diffuse and blurry diffracted beams and refinement of a disordered structure to rather poor *R*-values. It is noteworthy that the corresponding dimethylacetyl derivative in the *D*-glucopyranose series also showed some disorder in its crystalline form. It is also of interest that in both the *D*-glucopyranose and benzene series, we were unable to crystallize the propionyl derivatives.

Compound 1 was prepared by the acylation of 1,3,5-trihydroxybenzene with trimethylacetyl chloride and formed crystals with molecules arranged in interacting pairs by overlap of the arene rings about a centre of symmetry. C(2) lies over the centre of the opposing ring and is 3.320 (1) Å from the mean-plane of that ring, and C(1) overlaps C(3*^a^*) at a distance of 3.326 (2) Å. H(13*Bb*) is directed towards the centre of the C_6_ ring from the opposite side and is displaced 2.81 Å from the ring plane; the six H···C distances lie in the range 3.06–3.22 Å; supercripts denote symmetry operations. In contrast to 1,3,5-triacetoxybenzene (2) (previous paper, Haines and Hughes, 2009), there are no intermolecular C—H···O contacts with H···O distances less than 2.55 Å. There are also differences in intramolecular structure from that in 2 in the arrangement of carbonyl groups around the ring. Thus, the three acetoxy groups are essentially planar (as in 2) but have normals rotated -57.75 (4), -62.36 (4) and 63.36 (4)° from the normal to the mean-plane of the C_6_ ring (in contrast to the three positive angles in 2). Also, the carbonyl O-atoms of two groups here are 'up' and one is 'down' (in contrast to three 'up' in 2), and the propeller-style arrangement of substituent groups is spoiled with the plane of the 'down' group twisted in the opposite direction. In both compounds, all the C_ring_—O—C—*R* (*R*=Me or CMe_3_) conformations are *trans*. Dimensions are available in the archived CIFs.

### Experimental

The title compound was prepared by conventional acylation of the parent 1,3,5-trihydroxybenzene and gave analytical data (HRMS) and spectral data (^1^H and ^13^C NMR) in full accord with the expected structure.

*1,3,5-Tris-(trimethylacetoxy)benzene* (1) - 1,3,5-Trihydroxybenzene (0.63 g) was dissolved in pyridine (6 ml), trimethylacetyl chloride (3.7 ml) was added, and the mixture was then stored for 12 h at room temperature. Water (1 ml) was added to destroy excess acyl chloride, and the mixture was then poured on to ice, affording a sticky solid which was separated and dissolved in dichloromethane (30 ml). This solution was washed with saturated aqueous sodium hydrogen carbonate, water, and then dried over anhydrous sodium sulfate. Concentration of the filtered solution gave a crystalline solid which was recrystallized from ethanol to give compound 1 (0.947 g, 50%), m.p. 163–165 °C; δ_H_(CDCl_3_) 6.77, (s, 3H), 1.33 (s, 27H); δ_C_ (CDCl_3_) 176.43, 151.68, 112.57, 39.02, 26.91. *m/z* (ES): 396.2 [*M*+NH_4_]^+^. (Found: [*M*+NH_4_]^+^ 396.2381. C_21_H_34_NO_6_ requires *m/z* 396.2381).

### Refinement

Hydrogen atoms were included in idealized positions and their *U*_iso_ values were set to ride on the *U*_eq_ values of the parent carbon atoms.

### Crystal data

**Table:**

C_21_H_30_O_6_	*V* = 1068.87 (5) Å^3^
*M**_r_* = 378.45	*Z* = 2
Triclinic, *P*1	*F*(000) = 408
Hall symbol: -P 1	*D*_x_ = 1.176 Mg m^−^^3^
*a* = 9.4812 (3) Å	Mo *K*α radiation, λ = 0.71073 Å
*b* = 10.6885 (3) Å	µ = 0.09 mm^−^^1^
*c* = 11.4799 (3) Å	*T* = 140 K
α = 91.138 (2)°	Plate, colourless
β = 97.649 (3)°	0.65 × 0.32 × 0.15 mm
γ = 111.624 (3)°	

### Data collection

**Table:**

Oxford Diffraction Xcalibur 3/CCD diffractometer	6205 independent reflections
Radiation source: Enhance (Mo) X-ray Source	4605 reflections with *I* > 2σ(*I*)
graphite	*R*_int_ = 0.038
Detector resolution: 16.0050 pixels mm^-1^	θ_max_ = 30.0°, θ_min_ = 3.2°
Thin–slice φ and ω scans	*h* = −13→13
Absorption correction: multi-scan (*CrysAlis PRO* RED; Oxford Diffraction, 2008)	*k* = −15→15
*T*_min_ = 0.920, *T*_max_ = 1.059	*l* = −16→16
26503 measured reflections	

### Refinement

**Table:**

Refinement on *F*^2^	Primary atom site location: structure-invariant direct methods
Least-squares matrix: full	Secondary atom site location: difference Fourier map
*R*[*F*^2^ > 2σ(*F*^2^)] = 0.047	Hydrogen site location: inferred from neighbouring sites
*wR*(*F*^2^) = 0.132	H-atom parameters constrained
*S* = 1.08	*w* = 1/[σ^2^(*F*_o_^2^) + (0.0693*P*)^2^ + 0.1174*P*] where *P* = (*F*_o_^2^ + 2*F*_c_^2^)/3
6205 reflections	(Δ/σ)_max_ < 0.001
244 parameters	Δρ_max_ = 0.65 e Å^−^^3^
0 restraints	Δρ_min_ = −0.34 e Å^−^^3^

### Special details

**Table:**

Experimental. CrysAlisPro RED, Oxford Diffraction Ltd., Version 1.171.32.24 Empirical absorption correction using spherical harmonics, implemented in SCALE3 ABSPACK scaling algorithm.
Geometry. All e.s.d.'s (except the e.s.d. in the dihedral angle between two l.s. planes) are estimated using the full covariance matrix. The cell e.s.d.'s are taken into account individually in the estimation of e.s.d.'s in distances, angles and torsion angles; correlations between e.s.d.'s in cell parameters are only used when they are defined by crystal symmetry. An approximate (isotropic) treatment of cell e.s.d.'s is used for estimating e.s.d.'s involving l.s. planes.
Refinement. Refinement of *F*^2^ against ALL reflections. The weighted *R*-factor *wR* and goodness of fit *S* are based on *F*^2^, conventional *R*-factors *R* are based on *F*, with *F* set to zero for negative *F*^2^. The threshold expression of *F*^2^ > σ(*F*^2^) is used only for calculating *R*-factors(gt) *etc*. and is not relevant to the choice of reflections for refinement. *R*-factors based on *F*^2^ are statistically about twice as large as those based on *F*, and *R*- factors based on ALL data will be even larger.

### Fractional atomic coordinates and isotropic or equivalent isotropic displacement parameters (Å^2^)

**Table:**

	*x*	*y*	*z*	*U*_iso_*/*U*_eq_	
C1	0.27936 (13)	0.48381 (12)	0.50299 (10)	0.0183 (2)	
C2	0.39408 (13)	0.61026 (12)	0.50434 (10)	0.0187 (2)	
H2	0.3990	0.6620	0.4397	0.022*	
C3	0.50064 (13)	0.65635 (11)	0.60527 (10)	0.0185 (2)	
C4	0.49732 (13)	0.58123 (12)	0.70237 (10)	0.0193 (2)	
H4	0.5708	0.6140	0.7693	0.023*	
C5	0.38065 (13)	0.45569 (12)	0.69610 (10)	0.0183 (2)	
C6	0.26969 (13)	0.40486 (12)	0.59784 (10)	0.0191 (2)	
H6	0.1914	0.3206	0.5956	0.023*	
O1	0.17567 (10)	0.42434 (8)	0.40037 (7)	0.02236 (19)	
C11	0.08728 (12)	0.49094 (12)	0.34944 (10)	0.0182 (2)	
O11	0.08760 (11)	0.59371 (9)	0.39219 (8)	0.0276 (2)	
C12	−0.01207 (13)	0.41230 (12)	0.23672 (10)	0.0194 (2)	
C13	−0.14944 (15)	0.29840 (14)	0.27447 (13)	0.0293 (3)	
H13A	−0.2155	0.2458	0.2058	0.044*	
H13B	−0.2056	0.3368	0.3178	0.044*	
H13C	−0.1129	0.2416	0.3234	0.044*	
C14	0.07752 (16)	0.35349 (16)	0.16576 (12)	0.0313 (3)	
H14A	0.0122	0.3041	0.0955	0.047*	
H14B	0.1123	0.2940	0.2125	0.047*	
H14C	0.1644	0.4254	0.1447	0.047*	
C15	−0.06716 (15)	0.50707 (14)	0.16204 (11)	0.0272 (3)	
H15A	−0.1303	0.4582	0.0908	0.041*	
H15B	0.0199	0.5802	0.1427	0.041*	
H15C	−0.1257	0.5423	0.2057	0.041*	
O3	0.62184 (10)	0.78028 (9)	0.60186 (8)	0.0246 (2)	
C31	0.63776 (14)	0.88421 (12)	0.67938 (11)	0.0236 (2)	
O31	0.55553 (14)	0.87315 (11)	0.75191 (10)	0.0443 (3)	
C32	0.77047 (16)	1.01112 (13)	0.65958 (12)	0.0296 (3)	
C33	0.9154 (2)	0.9794 (2)	0.6641 (3)	0.0782 (8)	
H33A	0.9998	1.0594	0.6518	0.117*	
H33B	0.8997	0.9103	0.6036	0.117*	
H33C	0.9379	0.9487	0.7397	0.117*	
C34	0.7314 (3)	1.05974 (18)	0.54077 (15)	0.0601 (6)	
H34A	0.8148	1.1401	0.5277	0.090*	
H34B	0.6400	1.0790	0.5397	0.090*	
H34C	0.7147	0.9909	0.4797	0.090*	
C35	0.7955 (3)	1.11854 (17)	0.75681 (16)	0.0604 (6)	
H35A	0.8793	1.1992	0.7450	0.091*	
H35B	0.8189	1.0867	0.8317	0.091*	
H35C	0.7040	1.1378	0.7553	0.091*	
O5	0.37241 (10)	0.38397 (9)	0.79761 (7)	0.02357 (19)	
C51	0.39004 (16)	0.26392 (14)	0.79296 (11)	0.0259 (3)	
O51	0.40977 (19)	0.21582 (13)	0.70459 (10)	0.0585 (4)	
C52	0.38396 (14)	0.20385 (13)	0.91138 (10)	0.0224 (2)	
C53	0.54114 (17)	0.27468 (17)	0.98640 (13)	0.0378 (3)	
H53A	0.5411	0.2383	1.0622	0.057*	
H53B	0.5625	0.3696	0.9963	0.057*	
H53C	0.6185	0.2606	0.9479	0.057*	
C54	0.26045 (17)	0.22568 (16)	0.97295 (12)	0.0328 (3)	
H54A	0.2584	0.1866	1.0476	0.049*	
H54B	0.1622	0.1835	0.9247	0.049*	
H54C	0.2833	0.3207	0.9852	0.049*	
C55	0.3493 (2)	0.05325 (15)	0.89167 (14)	0.0373 (3)	
H55A	0.3453	0.0137	0.9661	0.056*	
H55B	0.4285	0.0404	0.8547	0.056*	
H55C	0.2522	0.0108	0.8418	0.056*	

### Atomic displacement parameters (Å^2^)

**Table:**

	*U*^11^	*U*^22^	*U*^33^	*U*^12^	*U*^13^	*U*^23^
C1	0.0195 (5)	0.0191 (5)	0.0172 (5)	0.0095 (4)	−0.0006 (4)	−0.0011 (4)
C2	0.0228 (5)	0.0182 (5)	0.0171 (5)	0.0094 (4)	0.0044 (4)	0.0031 (4)
C3	0.0178 (5)	0.0159 (5)	0.0210 (5)	0.0047 (4)	0.0051 (4)	−0.0007 (4)
C4	0.0193 (5)	0.0216 (6)	0.0176 (5)	0.0090 (4)	0.0008 (4)	−0.0011 (4)
C5	0.0233 (5)	0.0192 (5)	0.0158 (5)	0.0112 (4)	0.0047 (4)	0.0040 (4)
C6	0.0199 (5)	0.0156 (5)	0.0215 (5)	0.0063 (4)	0.0028 (4)	0.0017 (4)
O1	0.0258 (4)	0.0196 (4)	0.0206 (4)	0.0107 (3)	−0.0062 (3)	−0.0025 (3)
C11	0.0172 (5)	0.0191 (5)	0.0181 (5)	0.0063 (4)	0.0028 (4)	0.0038 (4)
O11	0.0308 (5)	0.0224 (5)	0.0299 (5)	0.0139 (4)	−0.0048 (4)	−0.0043 (4)
C12	0.0195 (5)	0.0225 (6)	0.0164 (5)	0.0090 (4)	0.0002 (4)	0.0000 (4)
C13	0.0239 (6)	0.0247 (6)	0.0324 (7)	0.0024 (5)	0.0000 (5)	−0.0002 (5)
C14	0.0347 (7)	0.0423 (8)	0.0219 (6)	0.0220 (6)	0.0007 (5)	−0.0054 (5)
C15	0.0291 (6)	0.0305 (7)	0.0218 (6)	0.0131 (5)	−0.0027 (5)	0.0036 (5)
O3	0.0235 (4)	0.0191 (4)	0.0259 (4)	0.0005 (3)	0.0085 (3)	−0.0015 (3)
C31	0.0275 (6)	0.0184 (6)	0.0225 (6)	0.0065 (5)	0.0019 (5)	0.0025 (4)
O31	0.0604 (7)	0.0248 (5)	0.0460 (6)	0.0066 (5)	0.0302 (6)	−0.0023 (5)
C32	0.0330 (7)	0.0183 (6)	0.0305 (7)	0.0016 (5)	0.0037 (5)	0.0041 (5)
C33	0.0305 (9)	0.0388 (11)	0.153 (2)	−0.0027 (8)	0.0166 (12)	0.0135 (13)
C34	0.0894 (15)	0.0306 (9)	0.0339 (9)	−0.0049 (9)	−0.0011 (9)	0.0123 (7)
C35	0.0897 (15)	0.0251 (8)	0.0400 (9)	−0.0093 (9)	0.0119 (10)	−0.0039 (7)
O5	0.0359 (5)	0.0216 (4)	0.0175 (4)	0.0152 (4)	0.0052 (3)	0.0049 (3)
C51	0.0360 (7)	0.0269 (6)	0.0214 (6)	0.0181 (6)	0.0071 (5)	0.0054 (5)
O51	0.1225 (12)	0.0528 (8)	0.0307 (6)	0.0611 (8)	0.0299 (7)	0.0150 (5)
C52	0.0281 (6)	0.0241 (6)	0.0177 (5)	0.0128 (5)	0.0030 (4)	0.0057 (4)
C53	0.0311 (7)	0.0462 (9)	0.0302 (7)	0.0096 (6)	−0.0015 (6)	0.0051 (6)
C54	0.0387 (7)	0.0405 (8)	0.0267 (7)	0.0201 (7)	0.0140 (6)	0.0122 (6)
C55	0.0513 (9)	0.0267 (7)	0.0374 (8)	0.0178 (7)	0.0084 (7)	0.0069 (6)

### Geometric parameters (Å, °)

**Table:**

C1—C6	1.3829 (16)	C31—C32	1.5184 (18)
C1—C2	1.3861 (16)	C32—C35	1.516 (2)
C1—O1	1.3999 (14)	C32—C34	1.518 (2)
C2—C3	1.3802 (16)	C32—C33	1.527 (3)
C2—H2	0.9300	C33—H33A	0.9600
C3—C4	1.3836 (16)	C33—H33B	0.9600
C3—O3	1.4032 (14)	C33—H33C	0.9600
C4—C5	1.3822 (17)	C34—H34A	0.9600
C4—H4	0.9300	C34—H34B	0.9600
C5—C6	1.3830 (16)	C34—H34C	0.9600
C5—O5	1.4025 (13)	C35—H35A	0.9600
C6—H6	0.9300	C35—H35B	0.9600
O1—C11	1.3699 (13)	C35—H35C	0.9600
C11—O11	1.1931 (14)	O5—C51	1.3546 (15)
C11—C12	1.5221 (16)	C51—O51	1.1955 (16)
C12—C14	1.5289 (17)	C51—C52	1.5139 (17)
C12—C15	1.5294 (17)	C52—C55	1.5251 (19)
C12—C13	1.5391 (17)	C52—C53	1.5286 (19)
C13—H13A	0.9600	C52—C54	1.5298 (17)
C13—H13B	0.9600	C53—H53A	0.9600
C13—H13C	0.9600	C53—H53B	0.9600
C14—H14A	0.9600	C53—H53C	0.9600
C14—H14B	0.9600	C54—H54A	0.9600
C14—H14C	0.9600	C54—H54B	0.9600
C15—H15A	0.9600	C54—H54C	0.9600
C15—H15B	0.9600	C55—H55A	0.9600
C15—H15C	0.9600	C55—H55B	0.9600
O3—C31	1.3601 (15)	C55—H55C	0.9600
C31—O31	1.1933 (16)		
			
C6—C1—C2	122.36 (11)	C35—C32—C31	108.92 (12)
C6—C1—O1	116.42 (10)	C34—C32—C31	108.98 (12)
C2—C1—O1	120.95 (10)	C35—C32—C33	109.08 (17)
C3—C2—C1	117.42 (10)	C34—C32—C33	110.80 (18)
C3—C2—H2	121.3	C31—C32—C33	109.09 (12)
C1—C2—H2	121.3	C32—C33—H33A	109.5
C2—C3—C4	122.69 (11)	C32—C33—H33B	109.5
C2—C3—O3	116.51 (10)	H33A—C33—H33B	109.5
C4—C3—O3	120.61 (10)	C32—C33—H33C	109.5
C5—C4—C3	117.45 (10)	H33A—C33—H33C	109.5
C5—C4—H4	121.3	H33B—C33—H33C	109.5
C3—C4—H4	121.3	C32—C34—H34A	109.5
C4—C5—C6	122.43 (10)	C32—C34—H34B	109.5
C4—C5—O5	117.03 (10)	H34A—C34—H34B	109.5
C6—C5—O5	120.41 (10)	C32—C34—H34C	109.5
C1—C6—C5	117.64 (11)	H34A—C34—H34C	109.5
C1—C6—H6	121.2	H34B—C34—H34C	109.5
C5—C6—H6	121.2	C32—C35—H35A	109.5
C11—O1—C1	118.78 (9)	C32—C35—H35B	109.5
O11—C11—O1	123.06 (11)	H35A—C35—H35B	109.5
O11—C11—C12	126.51 (10)	C32—C35—H35C	109.5
O1—C11—C12	110.38 (9)	H35A—C35—H35C	109.5
C11—C12—C14	111.20 (10)	H35B—C35—H35C	109.5
C11—C12—C15	108.90 (10)	C51—O5—C5	119.00 (9)
C14—C12—C15	109.33 (10)	O51—C51—O5	122.18 (12)
C11—C12—C13	106.60 (10)	O51—C51—C52	126.14 (12)
C14—C12—C13	110.37 (11)	O5—C51—C52	111.67 (10)
C15—C12—C13	110.40 (10)	C51—C52—C55	108.46 (11)
C12—C13—H13A	109.5	C51—C52—C53	107.48 (11)
C12—C13—H13B	109.5	C55—C52—C53	110.45 (12)
H13A—C13—H13B	109.5	C51—C52—C54	110.91 (10)
C12—C13—H13C	109.5	C55—C52—C54	109.98 (12)
H13A—C13—H13C	109.5	C53—C52—C54	109.53 (11)
H13B—C13—H13C	109.5	C52—C53—H53A	109.5
C12—C14—H14A	109.5	C52—C53—H53B	109.5
C12—C14—H14B	109.5	H53A—C53—H53B	109.5
H14A—C14—H14B	109.5	C52—C53—H53C	109.5
C12—C14—H14C	109.5	H53A—C53—H53C	109.5
H14A—C14—H14C	109.5	H53B—C53—H53C	109.5
H14B—C14—H14C	109.5	C52—C54—H54A	109.5
C12—C15—H15A	109.5	C52—C54—H54B	109.5
C12—C15—H15B	109.5	H54A—C54—H54B	109.5
H15A—C15—H15B	109.5	C52—C54—H54C	109.5
C12—C15—H15C	109.5	H54A—C54—H54C	109.5
H15A—C15—H15C	109.5	H54B—C54—H54C	109.5
H15B—C15—H15C	109.5	C52—C55—H55A	109.5
C31—O3—C3	118.41 (9)	C52—C55—H55B	109.5
O31—C31—O3	122.71 (12)	H55A—C55—H55B	109.5
O31—C31—C32	126.27 (12)	C52—C55—H55C	109.5
O3—C31—C32	111.01 (11)	H55A—C55—H55C	109.5
C35—C32—C34	109.94 (14)	H55B—C55—H55C	109.5
			
C6—C1—C2—C3	−0.10 (17)	O1—C11—C12—C13	79.48 (11)
O1—C1—C2—C3	−173.88 (10)	C2—C3—O3—C31	118.90 (12)
C1—C2—C3—C4	0.68 (17)	C4—C3—O3—C31	−65.99 (14)
C1—C2—C3—O3	175.68 (9)	C3—O3—C31—O31	2.02 (18)
C2—C3—C4—C5	−0.51 (17)	C3—O3—C31—C32	−177.74 (10)
O3—C3—C4—C5	−175.32 (10)	O31—C31—C32—C35	7.0 (2)
C3—C4—C5—C6	−0.24 (17)	O3—C31—C32—C35	−173.27 (13)
C3—C4—C5—O5	−176.02 (10)	O31—C31—C32—C34	−112.97 (18)
C2—C1—C6—C5	−0.59 (17)	O3—C31—C32—C34	66.78 (16)
O1—C1—C6—C5	173.45 (10)	O31—C31—C32—C33	125.94 (19)
C4—C5—C6—C1	0.77 (17)	O3—C31—C32—C33	−54.31 (18)
O5—C5—C6—C1	176.41 (10)	C4—C5—O5—C51	−117.71 (12)
C6—C1—O1—C11	127.36 (11)	C6—C5—O5—C51	66.42 (15)
C2—C1—O1—C11	−58.51 (14)	C5—O5—C51—O51	−1.3 (2)
C1—O1—C11—O11	−4.65 (16)	C5—O5—C51—C52	177.76 (10)
C1—O1—C11—C12	177.63 (10)	O51—C51—C52—C55	−20.2 (2)
O11—C11—C12—C14	141.50 (13)	O5—C51—C52—C55	160.80 (12)
O1—C11—C12—C14	−40.87 (13)	O51—C51—C52—C53	99.26 (18)
O11—C11—C12—C15	20.96 (17)	O5—C51—C52—C53	−79.78 (14)
O1—C11—C12—C15	−161.40 (9)	O51—C51—C52—C54	−141.04 (17)
O11—C11—C12—C13	−98.15 (14)	O5—C51—C52—C54	39.92 (15)

### Hydrogen-bond geometry (Å, °)

**Table:**

*D*—H···*A*	*D*—H	H···*A*	*D*···*A*	*D*—H···*A*
C13—H13B···Cg1^i^	0.96	2.82	3.7608 (16)	168

Symmetry codes: (i) −*x*, −*y*+1, −*z*+1.
